# Supplementary material for: Diverse viral proteases activate the NLRP1 inflammasome
Source: eLife. 2021 Jan 7;10:e60609. doi: 10.7554/eLife.60609 (PMC7857732; doi:10.7554/eLife.60609)
Supplement: Transparent reporting form [file elife-60609-transrepform.doc]

***eLife’s* transparent reporting form**

We encourage authors to provide detailed information *within their submission* to facilitate the interpretation and replication of experiments. Authors can upload supporting documentation to indicate the use of appropriate reporting guidelines for health-related research (see [EQUATOR Network](http://www.equator-network.org/ )), life science research (see the [BioSharing Information Resource](https://biosharing.org/)), or the [ARRIVE guidelines](http://www.plosbiology.org/article/info:doi/10.1371/journal.pbio.1000412) for reporting work involving animal research. Where applicable, authors should refer to any relevant reporting standards documents in this form.

If you have any questions, please consult our Journal Policies and/or contact us: [editorial@elifesciences.org](mailto:editorial@elifesciences.org).

**Sample-size estimation**

- You should state whether an appropriate sample size was computed when the study was being designed
- You should state the statistical method of sample size computation and any required assumptions
- If no explicit power analysis was used, you should describe how you decided what sample (replicate) size (number) to use

Please outline where this information can be found within the submission (e.g., sections or figure legends), or explain why this information doesn’t apply to your submission:

Statistical tests are given in every figure legend. The conditions in each HEK-Blue IL-1β assay were done in triplicate, as stated in the “Materials and Methods” section. Because of the consistency of the data, no explicit power analysis was used.

**Replicates**

- You should report how often each experiment was performed
- You should include a definition of biological versus technical replication
- The data obtained should be provided and sufficient information should be provided to indicate the number of independent biological and/or technical replicates
- If you encountered any outliers, you should describe how these were handled
- Criteria for exclusion/inclusion of data should be clearly stated
- High-throughput sequence data should be uploaded before submission, with a private link for reviewers provided (these are available from both GEO and ArrayExpress)

Please outline where this information can be found within the submission (e.g., sections or figure legends), or explain why this information doesn’t apply to your submission:

-The conditions in each HEK-Blue IL-1 β assay were done in triplicate, as stated in the “Materials and Methods” section. We define these as biological replicates because each assay data point represents an independent measurement beginning with independent transfections for all replicates. The individual data values are shown within each graph.

-No outliers were excluded.

**Statistical reporting**

- Statistical analysis methods should be described and justified
- Raw data should be presented in figures whenever informative to do so (typically when N per group is less than 10)
- For each experiment, you should identify the statistical tests used, exact values of N, definitions of center, methods of multiple test correction, and dispersion and precision measures (e.g., mean, median, SD, SEM, confidence intervals; and, for the major substantive results, a measure of effect size (e.g., Pearson's r, Cohen's d)
- Report exact p-values wherever possible alongside the summary statistics and 95% confidence intervals. These should be reported for all key questions and not only when the p-value is less than 0.05.

Please outline where this information can be found within the submission (e.g., sections or figure legends), or explain why this information doesn’t apply to your submission:

Statistical reporting is relevant for the HEK-Blue IL-1 β assay described in the “Materials and Methods” section. Statistical tests are given in every figure legend. The individual data points are shown within each scatter and bar graph. The height of the bar represents the mean, with extended lines to represent the mean ± SD. Because there are more than three groups and the group means in each HEK-Blue experiment were determined to be significantly different (P<0.0001) by either ordinary one-way ANOVA or ordinary two-way ANOVA (described in the figure legends), we conducted ANOVA post-tests to determine which group means differed. For all graphs, conditions with statistical significance for the post-test (described in the figure legends), as indicated by asterisks, are presented relative to their respective controls. P < 0.05 was considered to be statistically significant. ** P < 0.01, *** P < 0.001, **** P < 0.0001. Statistical analyses were performed using GraphPad Prism 8 (Graphpad software Inc.).

With the exception of the exact p-value reported for EV71 activation of human NLRP1 in Figure 4C, p-values that were not significant are not reported.

(For large datasets, or papers with a very large number of statistical tests, you may upload a single table file with tests, Ns, etc., with reference to sections in the manuscript.)

**Group allocation**

- Indicate how samples were allocated into experimental groups (in the case of clinical studies, please specify allocation to treatment method); if randomization was used, please also state if restricted randomization was applied
- Indicate if masking was used during group allocation, data collection and/or data analysis

Please outline where this information can be found within the submission (e.g., sections or figure legends), or explain why this information doesn’t apply to your submission:

Group allocation is not applicable to our study.

**Additional data files (“source data”)**

- We encourage you to upload relevant additional data files, such as numerical data that are represented as a graph in a figure, or as a summary table
- Where provided, these should be in the most useful format, and they can be uploaded as “Source data” files linked to a main figure or table
- Include model definition files including the full list of parameters used
- Include code used for data analysis (e.g., R, MatLab)
- Avoid stating that data files are “available upon request”

Please indicate the figures or tables for which source data files have been provided:

To facilitate reproducibility of the motif generation and search, all viral polyprotein sequences used for motif generation and benchmarking the motif are found in **Supplemental File 1** and **Supplemental File 2**. The 8-mer cleavage sites within each polyprotein determined by alignment to RefSeq enteroviral polyproteins are also reported with the exception of the VP3-VP1 site within enterovirus D polyproteins (See “Materials and Methods”). Additionally, the FIMO-generated (MEME Suite) p-values from motif searching against viral polyproteins reported in **Supplemental File 1** and the human cleavage sites reported in the Laitinen et al 2016 dataset are reported in **Supplemental File 3** and **Supplemental File 4**.
